# Care trajectories of chronically ill older adult patients discharged from hospital: a quantitative cross-sectional study using health insurance claims data

**DOI:** 10.1186/s12877-019-1302-0

**Published:** 2019-10-15

**Authors:** Yvonne de Man, Femke Atsma, Wilma Jonkers, Sophia E. J. A. de Rooij, Gert P. Westert, Patrick P. T. Jeurissen, A. Stef Groenewoud

**Affiliations:** 1 Radboud university medical center, Radboud Institute for Health Sciences, IQ healthcare, P.O. Box 9101, 114, 6500 HB Nijmegen, the Netherlands; 2 Division of Health Care, Achmea Insurances, Zwolle, The Netherlands; 3 University of Groningen, University Medical Center Groningen, University Center for Geriatric Medicine, Groningen, The Netherlands; 4 AMC, Department of Internal Medicine, Geriatrics, Amsterdam, NL The Netherlands

**Keywords:** Healthcare utilization, Aftercare, Home and community based care and services, Healthcare policy, Hospital/ambulatory care, Long-term care, Hip fracture, Quantitative research methods

## Abstract

**Background:**

For older adults, a good transition from hospital to the primary or long-term care setting can decrease readmissions. This paper presents the 6-month post-discharge healthcare utilization of older adults and describes the numbers of readmissions and deaths for the most frequently occurring aftercare arrangements as a starting point in optimizing the post-discharge healthcare organization.

**Methods:**

This cross-sectional study included older adults insured with the largest Dutch insurance company. We described the utilization of healthcare within 180 days after discharge from their first hospital admission of 2015 and the most frequently occurring combinations of aftercare in the form of geriatric rehabilitation, community nursing, long-term care, and short stay during the first 90 days after discharge. We calculated the proportion of older adults that was readmitted or had died in the 90–180 days after discharge for the six most frequent combinations. We performed all analyses in the total group of older adults and in a sub-group of older adults who had been hospitalized due to a hip fracture.

**Results:**

A total of 31.7% of all older adults and 11.4% of the older adults with a hip fracture did not receive aftercare. Almost half of all older adults received care of a community nurse, whereas less than 5% received long-term home care. Up to 18% received care in a nursing home during the 6 months after discharge. Readmissions were lowest for older adults with a short stay and highest in the group geriatric rehabilitation + community nursing. Mortality was lowest in the total group of older aldults and subgroup with hip fracture without aftercare.

**Conclusions:**

The organization of post-discharge healthcare for older adults may not be organized sufficiently to guarantee appropriate care to restore functional activity. Although receiving aftercare is not a clear predictor of readmissions in our study, the results do seem to indicate that older adults receiving community nursing in the first 90 days less often die compared to older adults with other types of aftercare or no aftercare. Future research is necessary to examine predictors of readmissions and mortality in both older adult patients discharged from hospital.

## Background

Approximately 18% of people over 75 years old are newly admitted to the hospital annually [1]. These hospitalizations bring substantial risks,. Among older adults who are clinically admitted, irreversible loss of function can arise (this number varies between 30 to 60%) [2]. These older adult patients are especially at risk of becoming frail. Frail older adult patients are less capable to live independently at home, leading to a greater dependency in daily life, the loss of the ability to care for oneself, an increased mortality risk [3, 4] and an increased healthcare utilization in different care settings [5]. One study reported that a third of frail older adults die within 100 days after discharge from hospital [6].

Research shows that a good transition from hospital to the primary or long-term care setting can decrease readmission rates [7]. However, we currently lack insight in the type and intensity of care that most older adult patients actually receive after a clinical admission and whether this is optimally organized in order to prevent adverse outcomes. Insight into the healthcare utilization of a patient group forms the first crucial step toward improving their healthcare, and is a tool for the identification of potential areas for improvement. This paper therefore aims to present the 6-month post-discharge healthcare utilization of older adult patients and describe the numbers of readmissions and deaths for the most frequently occurring aftercare arrangements as a starting point in optimizing the post-discharge healthcare organization.

We hypothesize that older adults with any type of intensive aftercare would have lower mortality and readmission rates than chronically ill older adult patients who are discharged otherwise.

## Methods

### Design

The design of this study is an observational cross-sectional study using health insurance claims data in the period of January 2015–July 2016. Quantitative measures were used in order to gain insight into the healthcare utilization of older adults after their hospital admission and describe the numbers of readmissions and deaths.

### Population

We included subjects insured by the largest Dutch insurance company (30% market share) with at least one claimed hospital admission in 2015. We selected all patients who were living in one of the company’s care-office regions in 2015. Hence, we were able to analyze the full chain of healthcare, including hospital care, general practitioners care, community nursing, medications, and long-term care. We attempted to define frail older adult patients from our dataset as they are most at risk for adverse events.

Research done by Makai et al. has shown that age, sex, medication use, and being incontinent are predicting variables for frailty when analyzing claims data [8]*.* We therefore included patients if they were aged 75 and up, used medications from at least two pharmacy-based cost groups [9], which are defined by The National Health Care Institute [10] and used incontinence materials in 2015.

Of the 375,044 insurees that were 75 years or older in 2015, 247,529 (66%) were clinically admitted to a hospital. With the additional criteria, we identified 21,258 (8.7%) as likely to be frail. This is much lower than the percentages of frailty found in the literature [11] and underpins the belief that the best way to identify frail older adults is by performing a comprehensive geriatric assessment [12, 13]. Thus, in order to not confuse existing literature we abstain from using the term ‘frail’ for our subjects and will refer to them as chronically ill older adult patients.

We identified 21,258 hospitalized chronically ill older adult patients. They were clinically admitted at least once in that year to one of 96 different organizations: 87 hospitals and 9 private clinics. In order to obtain a more homogenous group, we also conducted all analyses in a subsample of our selection. We chose older adults clinically admitted with a hip fracture, as this is a typical acute event requiring hospitalization and rehabilitation.

### Data

The insurance company’s database contains all claims data and underlying care provided at the level of healthcare activities and performance codes of their insurees. The data also includes information about the healthcare providers, as well as the 4-digit postal codes, age, sex, and marital status of insurees.

Data on socioeconomic status (SES) of 2014 were retrieved from the Social and Cultural Planning Office (SCP) and linked by four digital postal codes to the claims data. SCP calculated social economic status scores based on information regarding education, income, and position in the labor market [14]. The scores resemble the social status of a postal code region, relative to the other regions in the Netherlands.

### Procedures and definitions

For each insuree, all claims under the healthcare law and long-term care act were recovered. Claims under the social support act were not included (these are reimbursed through municipalities). We identified the last day of a patient’s first admission in 2015. These included clinical admissions, intensive care unit (ICU), and emergency room (ER) contacts in a hospital or independent treatment centre. The claims database does not include ICD-10 codes. In order to determine what the underlying reason for admission was, the healthcare insurance company provided us with a reference table where each diagnosis-related group (DRG) was linked to a certain chapter of the ICD-10 codes. We selected all healthcare activities that were registered within a 180 day period after their discharge. As there are thousands of different healthcare activities, we clustered the data according to predefined meaningful categories [15] .

In order to describe the number of deaths and readmissions for different combinations of aftercare, we labeled chronically ill older adult patients according to the type(s) of care they received in the 90 days after admission: geriatric rehabilitation (GR), community nursing (CN), a short stay in a nursing home (ST), and long-term care (LTC). Geriatric rehabilitation (GR) is short-term and intensive care given by a multidisciplinary team either in a hospital ward or a nursing facility [16]. A person is eligible for GR when they can return home after this treatment. CN is nursing care at home for patients with an illness or disability. The aim is to ensure that the older adults can stay at home for as long as possible. A short stay (ST) is a temporary stay in a nursing home where people after hospitalization can stay for up to 12 weeks. Patients are eligible for an ST if they need more care than can be provided in the home situation [17]. LTC is intensive care provided at home or in a facility and is only for patients that will need permanent care 24/7. Patients with LTC pay an income-bound contribution to the costs [18].

We chose these four categories, as these are the types of usual aftercare that we believe might ward off readmissions. It was possible for each chronically ill older adult patient to be labeled with more than one category, resulting in multiple combinations of aftercare. For the six most frequently occurring combinations of aftercare, the group without any of these four types and the remaining patients (others) we identified subject characteristics and the readmission and mortality rates in the 90 days and 90–180 days after admission.

### Analyses

For both samples, means and standard deviations for age; median and ranges for SES, total 2015 spending, and number of morbidities; frequencies and percentages for sex, marital status, types of morbidities, and the three most frequently registered DRGs opened at admission, were calculated. We analyzed the six-month post-discharge healthcare utilization for all FE and FE admitted due to a hip fracture, for hospital care, GP care, long-term care, and community nursing care. We did this by calculating the number and percentage of chronically ill older adult patients with a certain type of treatment and by calculating the average number of treatments per user. To assess group differences, a T-test and a non-parametric equivalent were performed on all continuous variables. A Chi-square test was performed on all categorical variables.

All analyses were performed in SAS Enterprise Guide.

## Results

### Healthcare utilization profiles

In Table 1, descriptive statistics for all included chronically ill older adult patients are presented. The average age at the time of admission was 83.8 years and more females than males were clinically admitted (62.1%). The most common morbidities were hypertension (88.1%), heart conditions (62.4%), and high cholesterol (54.8%). Of all 21,258 chronically ill older adult patients, 505 (2.4%) died during their first 2015 admission, resulting in a total of 20,753 discharged patients. A total of 12.5% died during admission or during the 90 days after discharge. The five most common reasons for a hospital admission was injury or poisoning (7.0%), ischemia, CABG, PCI (6.2%), cerebrovascular diseases (5.3), diseases of the digestive system (5.2%), and cardiac arrhythmia (5.5%).

**Table 1:** Baseline characteristics

	Older adult with any admission in 2015 (*N* = 21,258)			Older adult admitted with hip fracture (*N* = 814)		
Measure	N (%)	Mean (SD)	Median [Range]	N	Mean (SD)	median
Age	21,258	83.8 (5.6)	83 [75–104]	814	86.7 (5.5)^*** c^	87 [75–101]
Male	8066 (37.9)			200 (24.6)^***^		
SES	21,258	−0.4 (1.2)	−0.2 [−5.7–2.8]	811	−0.3 (1.2) ^d^	− 0.1 [− 5.7–2.6]
Mortality during admission	505 (2.4)			23 (2.8)		
Total 2015 spending, €	21,258	24,793 (20,506)	19,045 [705–468,772]	814	30,283 (18,354)^***d^	27,054 [1424-177,666]
Marital status						
Married	6216 (29.2)			160 (19.7)		
Divorced/never been married	2616 (12.3)			101 (12.4)		
Widow (er)	6031 (28.4)			292 (35.9)		
Unknown	6395 (30.1)			261 (32.1)		
Number of morbidities^a^	21,258	3.8 (1.5)	4 [2–11]	814	3.3 (1.3) ^***d^	3 [2–8]
Hypertension	18,721 (88.1)			678 (83.3)^***^		
Heart conditions	13,266 (62.4)			466 (57.3)^*^		
High cholesterol levels	11,653 (54.8)			368 (45.2)^***^		
Asthma	6420 (30.2)			175 (21.5)^***^		
Chronic pain^b^	5639 (26.5)			215 (26.4)		
Diabetes Type 2	5638 (26.5)			190 (23.3)^*^		
COPD/heavy asthma	4846 (22.8)			124 (15.2)^***^		
Depression	4206 (19.8)			178 (21.9)		
Psychosis/alzheimers/addiction	3396 (16.0)			185 (22.7)^***^		
Diabetes Type 1	3224 (15.2)			72 (8.9)^***^		

All group differences were tested with a Chi-square test unless specified otherwise. ^a^Based on pharmaceutical cost groups. Only the top ten comorbidities are displayed, ^b^ Excluding opiods, ^c^ T-Test, ^d^ Non-parametric T-test (Mann-Whitney U Test). Differences are significant at the significance levels .001 (^***^), .01(^**^), and .05 (^*^)

Table 2 described the post-discharge healthcare utilization. During the six-month period after admission, 51.5% of all chronically ill older adult patients had a (non-ICU) readmission with a median number of hospital days of 12. Within the same time-frame, 12.3% of all patients were admitted to the ER with an average visit of 1.3 times (median 1). Almost half of all chronically ill older adult patients received care of a community nurse, whereas less than 5% received long-term home care. Up to 18% received care in a nursing home during the 6 months after discharge. Almost all patients visited their GP at least once, with a median of 7 visits, and in two thirds of the cases, the GP visited the patients at home (median of 3).

**Table 2:** The 180 day post-discharge healthcare utilization

	Older adult with any admission in 2015 (*N* = 21,258)			Older adult admitted with hip fracture (*N* = 814)		
Measure	N (%)	Mean (SD)	Median [Range]	N (%)	Mean (SD)	Median [Range]
Hospital						
Clinical admission	10,955 (51.5)	24.4 (29.2)	12 [1–179]	608 (74.7)^***^	44.9 (35.1)	38 [1–179]
ICU day	1487 (7.0)	1.0 (0.2)	1 [1–2]	34 (4.2)	1.0 (0)	1 [1–1]
ER visit	2612 (12.3)	1.3 (0.7)	1 [1–9]	60 (7.4)	1.2 (0.4)	1 [1–2]
Outpatient visit	17,540 (82.5)	4.8 (4.0)	4 [1–74]	660 (81.1)^***^	3.9 (3.3)	3 [1–22]
Day care treatment	2647 (12.5)	1.7 (2.4)	1 [1–77]	49 (6.0)	1.4 (0.7)	1 [1–4]
Surgery	4510 (21.2)	1.5 (0.9)	1 [1–14]	204 (25.1)^*^	1.3 (0.6)	1 [1–5]
Laboratory tests	13,697 (64.4)	10.1 (7.4)	9 [1–48]	487 (59.8)	10.6 (7.7)	10 [1–38]
Pathology tests	167 (0.8)	1.0 (0.2)	1 [1–2]	< 10		
CT-scan	4577 (21.5)	1.4 (0.9)	1 [1–14]	103 (12.7)^***^	1.2 (0.5)	1 [1–4]
MRI-scan	1123 (5.3)	1.2 (0.5)	1 [1–7]	18 (2.2)	1.2 (0.4)	1 [1–2]
PET/CT-scan	348 (1.6)	1.1 (0.3)	1 [1–3]	< 10		
Other nuclear tests	427 (2.0)	1.3 (0.5)	1 [1–4]	< 10		
Other imaging	12,668 (59.6)	2.8 (2.7)	2 [1–39]	621 (76.3)	2.8 (2.3)	2 [1–21]
Invasive diagnostics	3057 (14.4)	1.4 (1.0)	1 [1–18]	58 (7.1)	1.3 (0.7)	1 [1–4]
Other non-invasive diagnostics	11,961 (56.3)	2.7 (2.6)	2 [1–42]	322 (39.6)^**^	1.8 (1.4)	1 [1–11]
Rehabilitation days	4224 (19.9)	29.2 (23.4)	25 [1–161]	499 (61.3)^***^	34.6 (21.9)	29 [1–155]
Community home care						
Nursing hours	9675 (45.5)	26.8 (55.5)	0 [0–8]	367 (45.1)	18.8 (34.2)	5.9 [0.2–351.9]
Care giving hours	10,216 (48.1)	94.3 (128.7)	0 [0–59]	377 (46.3)	81.8 (110.9)	56.5 [0.1–1431.5]
LTC at home or facility						
Nursing hours	439 (2.1)	1551.5 (2531.0)	665 [5–21,329]	14 (1.7)	392.9 (466.3)	203 [45–1528]
Care giving hours	676 (3.2)	6510.0 (6710.7)	4230 [15–43,063]	16 (2.0)	3458.1 (3802.1)	2261.5 [105–13,890]
HCI package 3, hours	388 (1.8)	108.7 (70.5)	122 [1–185]	31 (3.8)	114.8 (66.5)	119 [17–184]
HCI package 4, hours	1152 (5.4)	115.0 (62.1)	124 [1–285]	93 (11.4)^*^	102.5 (60.8)	110 [1–195]
HCI package 5, hours	832 (3.9)	110.9 (60.6)	117 [1–223]	90 (11.1)	111.0 (60.5)	107.5 [1–195]
HCI package 6, hours	1227 (5.8)	101.9 (62.2)	102 [1–208]	93 (11.4)	103.6 (59.9)	103 [2–208]
HCI package 7, hours	50 (0.2)	96.6 (63.5)	93 [1–184]	< 10		
HCI package 8, hours	24 (0.1)	105.9 (69.3)	132 [1–199]	< 10		
HCI package 9, hours	90 (0.4)	65.4 (52.4)	48 [1–181]	26 (3.2)	66.7 (51.6)	63.5 [6–173]
HCI package 10, hours	60 (0.3)	25.3 (32.7)	12 [1–166]	< 10		
Short stay nursing home	1037 (4.9)	43.4 (33.3)	38 [1–184]	44 (5.4)	45.3 (31.9)	45 [1–156]
GP care						
Consultations	19,276 (90.7)	8.1 (6.4)	7 [1–71]	707 (86.9)^**^	7.5 (6.1)	6 [1–40]
Home visits	13,851 (65.2)	4.1 (3.8)	3 [1–53]	543 (66.7)	3.9 (3.3)	3 [1–19]

All group differences were tested with a Chi-square test. Differences are significant at the significance levels .001 (^***^), .01(^**^), and .05 (^*^). The health care intensity packages are a predefined package of healthcare. The higher the package number, the more intense healthcare a person receives. It ranges from living with daily assistance and nursing care to intensive palliative care in a home

*CT-scan* computerized tomography scan, *ER* emergency room, *GP* general practitioner, *HCI* health care intensity, *ICU* intensive care unit, *LTC* long-term care, *MRI-scan* magnetic resonance imaging scan

Significant differences between the total group of chronically ill older adult patients and chronically ill older adult patients with a hip fracture are: patients with a hip fracture were on average 3 years older, they had a higher healthcare spending in 2015, and more patients were readmitted with almost double the amount of hospital days and more rehabilitation days. Patients suffering from a hip fracture had significantly less outpatient visits, non-invasive diagnostics, and GP consultations compared to the total group of chronically ill older adult patients.

### Aftercare combinations and outcomes

The 6 most frequent combinations of aftercare for GR, CN, ST, and LTC during the first 90 days are: CN, LTC, GR + CN, CN + LTC, GR, and ST. These groups are shown in Tables 3 and 4 together with the group of chronically ill older adult patients not receiving any of these four types and the remaining category ‘other’. Table 3 depicts the results for the total group of chronically ill older adult patients and Table 4 shows the results for the subgroup with hip fracture. The results from both tables will be discussed below.

**Table 3:** Characteristics, mortality and admission rates by type of care received during the 90 days after discharge, total group

*N* = 20,753		CN (*N* = 8110, 39.1%)	LTC (*N* = 2961, 14.3%)	GR + CN (*N* = 890, 4.3%)	CN + LTC (*N* = 666, 3.2%)	GR (*N* = 457, 2.2%)	ST (*N* = 157, 0.8%)	Other combination (*N* = 930, 4.5%)	None of the four types of aftercare (*N* = 6582, 31.7%)
Age, mean (SD)		83.7 (5.4)	87.1 (5.6)	84.0 (5.2)	85.5 (5.4)	83.1 (5.2)	85.4 (5.5)	85.6 (5.3)	81.6 (4.9)
Male, %		36.7	25.7	34.8	35.6	33.5	31.9	27.5	47.8
SES, median [range]		−0.2 [−5.7–2.7]	−0.2 [−5.7–2.4]	− 0.1 [− 5.7–2.6]	−0.1 [− 5.7–2.4]	−0.3 [−4–2.6]	−0.3 [−3.5–1.8]	−0.3 [− 5.7–2.6]	−0.2 [− 5.7–2.8]
Number of morbidities (PCG)		4 [2–11]	3 [2–10]	4 [2–11]	4 [2–9]	3 [2–8]	3 [2–7]	3 [2–9]	4 [2–10]
Total 2015 costs in Euro's, median		23,993 [2364-216,437.4]	14,415.9 [1426-394,268]	40,559 [2669-180,726]	22,653 [3662.4-147,844]	42,445 [1344-160,956]	20,613 [4370-107,046]	26,769 [2887-171,810]	12,525 [705–468,772]
Top 3 DRG’s opened on admission	1	Injury, poisoning and certain other consequences of external causes (6%)	Injury, poisoning and certain other consequences of external causes (11%)	Injury - fracture hip/upper leg (9%)	Clinical geriatrics – other (11%)	Injury - fracture hip/upper leg (11%)	Injury, poisoning and certain other consequences of external causes (15%)	Injury, poisoning and certain other consequences of external causes (11%)	Ischemia. CABG. PCI (9%)
	2	Ischemia. CABG. PCI (5%)	Clinical geriatrics – other (10%)	Injury, poisoning and certain other consequences of external causes (9%)	Injury, poisoning and certain other consequences of external causes (10%)	Cerebrovascular disease (9%)	Generic symptoms (8%)	Clinical geriatrics – other (10%)	Cardiac arrhythmia (7%)
	3	Diseases of the digestive system (5%)	Injury - fracture hipz/upper leg (7%)	Cerebrovascular disease (8%)	Cerebrovascular disease (5%)	Injury, poisoning and certain other consequences of external causes (8%)	Cerebrovascular disease (6%)	Injury - fracture hip/upper leg (7%)	Disorders of digestive system (6%)
Mortality, N (%)	0–90 days	770 (9.5)	563 (18.9)	44 (4.9)	88 (13.2)	47 (10.3)	50 (38.2)	118 (12.7)	480 (7.3)
	90–180 days	454 (5.6)	249 (8.4)	54 (6.1)	53 (8.0)	29 (6.3)	12 (7.6)	103 (11.0)	170 (2.6)
Age at death, mean (SD)		84.2 (5.6)	87.9 (5.6)	85.5 (5.5)	86.6 (5.2)	86.0 (5.4)	86.9 (5.4)	86.1 (5.5)	84.0 (5.7)
90–180 day readmission (clinical), N (%)		1602 (19.8)	414 (14.0)	197 (22.1)	114 (17.1)	99 (21.7)	20 (15.3)	138 (14.8)	1292 (19.6)
Top 3 DRG’s opened on readmission	1	Cardiac arrhythmia (5%)	Injury, poisoning and certain other consequences of external causes (10%)	Injury - fracture hip/upper leg (9%)	Clinical geriatrics – other (10%)	PAOD (10%)	Cardiac arrhythmia (21%)	Clinical geriatrics – other (16%)	Ischemia. CABG. PCI (8%)
	2	Infection – LRT (5%)	COPD (6%)	Cerebrovascular disease (6%)	COPD (9%)	Injury, poisoning and certain other consequences of external causes (9%)	Injury, poisoning and certain other consequences of external causes (14%)	Cerebrovascular disease (12%)	Malignant neoplasms of unrinary organs (6%)
	3	Ischemia. CABG. PCI (5%)	Clinical geriatrics – other (6%)	PAOD (6%)	Infection – LRT (8%)	Ischemia. CABG. PCI (9%)	Infectious diseases- other (8%)	Injury - fracture hip/upper leg (8%)	Cardiac arrhythmia (6%)

*GR* geriatric rehabilitation, *LTC* long-term home care, *CN* community nursing, *PAOD* peripheral arterial occlusive disease, *LRT* Lower respiratory tract

**Table 4:** Characteristics, mortality and admission rates by type of care received during the 90 days after discharge, hip fracture

*N* = 791	Just Community nursing (CN) (*N* = 243, 30.7%)	Just long-term care (LTC) (*N* = 229, 29.0%)	GR + CN (*N* = 82, 10.4%)	Just Geriatric rehabilitation (GR) (*N* = 51, 6.5%)	CN + LTC (*N* = 25, 3.2%)	Just short stay nursing home (*N* = 10, 1.3%)	Other combination (*N* = 69, 8.7%)	None of the four types of aftercare (*N* = 90, 11.4%)
Age, mean (SD)	85.5 (5.5)	88.1 (5.3)	86.7 (5.6)	85.7 (5.8)	88.3 (5.0)	86.6 (5.5)	87.6 (5.7)	85.2 (5.3)
Male, %	25.1	17.2	32.9	21.6	32.0	50.0	26.1	30.0
SES, median [range]	−0.1 [−4–2.4]	−0.1 [−4.0–2.3]	− 0.1 [−5.7–2.6]	−0.6 [−3.5–2.1]	−0.2 [−2.1–2.1]	−0.7 [−2.3–1]	−0.2 [− 3.0–2.1]	0.0 [− 3.3–2.3]
Number of morbidities (PCG)	3 [2–7]	3 [2–8]	3 [2–7]	3 [2–7]	3 [2–8]	3 [2–7]	2 [3–7]	3 [2–7]
Total 2015 costs in Euro's, median	30,708 [9143–177,666]	18,014 [4579–124,274]	36,152 [7101–141,344]	38,698 [17801–96,409]	18,966 [10752–62,922]	30,893 [6138–44,763]	30,382 [8556–140,141]	23,484 [1424–107,259]
Mortality, N(%)								
0–90 days	13 (5.3)	53 (24.0)	6 (7.3)	7 (13.7)	6 (24.0)	3 (30.0)	6 (8.7)	19 (21.1)
90–180 days	8 (3.3)	18 (8.1)	5 (6.1)	3 (5.9)	2 (8.0)	2 (20.0)	8 (11.6)	3 (3.3)
Age at death, mean (SD)	87.6 (6.4)	88.9 (5.6)	89.7 (5.4)	87.8 (7.0)	89.9 (4.2)	87.8 (5.8)	91.5 (4.5)	87.7 (5.2)
90–180 day readmission (clinical), N (%)	36 (14.8)	22 (10.0)	18 (22.0)	8 (15.7)	1 (4.0)	0 (0.0)	6 (8.7)	12 (13.3)

*GR* geriatric rehabilitation, *LTC* long-term home care, *CN* community nursing

#### Total group chronically ill older adult patients

Age and sex rates differ between the groups; chronically ill older adult patients with LTC are older and more often female compared to the patients in the other groups; patients without aftercare are youngest (Table 3). The majority of the discharged patients received CN (39.1%) and 14.3% received LTC in the first 90 days. Almost a third received no aftercare. Each of the other combinations was offered to less than 5% of the patients. Overall, more females received any of the six combinations of aftercare, whereas the distribution of male/female was more or less equal in the group without any of these combinations of care. People receiving GR or a combination of GR and CN were mostly admitted due to a hip fracture, cerebrovascular diseases or another injury or poisoning. Patients receiving LTC were mostly admitted due to injury/poisoning, clinical geriatrics (including dementia) or a hip fracture. Patients with none of the four types of aftercare were mostly admitted due to ischemia, CABG or PCI, cardiac arrhythmia, or disorders of the digestive system.

##### Mortality

Within the first 90 days after discharge, the number of deaths is highest for patients who had a short stay in a LTC facility (38.2%). In the 90–180 day period, this dramatically drops to less than 8% and with 11%, the number of deceased patients is the highest for the ‘other’ combination. The lowest mortality rates were seen in the CN and GR + CN groups. Patients with LTC have the highest age at death whereas patients without aftercare die youngest.

##### Readmissions

We observe the lowest number of 90–180 day readmissions for patients who had an ST or LTC. The highest clinical readmission rates are seen in the groups GR + CN and GR. These rates exclude admissions to the rehabilitation ward. These patients are most often readmitted due to a hip fracture, cerebrovascular disease, or peripheral arterial occlusive disease (PAOD).

#### Chronically ill older adult patients with hip fracture

The majority of the discharged patients who were admitted due to a hip fracture, received community nursing care (30.7%), 29% received LTC and 11.4% received no aftercare in the form of GR, CN, LTC or ST (Table 4). Again, the age and sex rates differ over the multiple categories.

##### Mortality

The highest 90–180 day mortality rates are seen in the ‘ST’ and ‘other’ groups. These patients are younger and have less morbidities compared to the other groups. The lowest mortality was seen in patients with CN or none of the four types of aftercare (both 3.3%).

##### Readmissions

The highest readmission rates are seen in the GR + CN group (22%). Patients with an ST or CN + LTC are less often readmitted compared to the other groups.

## Discussion

This paper aimed to describe the post-discharge healthcare trajectories of chronically ill older adult patients and describe the mortality and readmission rates for the most common 90 day-post-discharge care arrangements. Our results seem to indicate that chronically ill older adult patients with any admission or an admission due to hip fracture, who receive CN in the first 90 days, less often die compared to patients receiving other types or no type of aftercare. However, chronically ill older adult patients receiving these types of aftercare also have one of the highest readmission rates.

Our hypothesis, that older adults with any type of predefined aftercare would have less readmissions and lower mortality rates than older adults receiving no aftercare, is not confirmed. On the contrary, older adults without aftercare less often die within 180 days.

An explanation for this could be that this group is in lesser need of care compared to the other groups. They are on average younger, a higher proportion is male, and the reasons for admission differ from the other groups. Another explanation could be that these older adults rely on informal care from for instance their spouse. However, they die at a younger age compared to older adults that did receive any of these types of intense care. Also, a large proportion is readmitted which suggests that they are not better off per se.

Describing care trajectories is especially important when we want to find flaws in the continuity of care and ultimately reduce fragmentation and optimize care. Older adult patients often suffer from multiple conditions and therefore receive care from multiple providers. This means that there is potential danger of fragmentation of care. A seamless continuity of care is most at risk during the patients’ transition from an institutional care setting to the home [19]. Although causality cannot be established from these data, our results coincide with previous research, that shows that a good transfer to home care and the use of a community nurse could reduce deaths and improve functional outcomes in older adults [6].

Research also shows that especially older adult patients admitted due to heart failure or with functional disability, which can be expected after hip fracture, deserve extra attention when transferring from the hospital to the home setting, especially with regards to avoidable readmissions [20]. Unfortunately, we know that transfer of care after discharge to the primary or long-term care setting is not always regulated sufficiently to guarantee continuity of care [21] and that in order for care transition programs to be effective, they require a more tailor-made approach [22].

Our inclusion criteria led to the inclusion of a heterogeneous group, as we could, for instance, see from the different diagnoses at admission. Differences in morbidity make it difficult to interpret the results. With our subgroup analyses we shed more light onthis issue. Post-discharge healthcare utilization for chronically ill older adult patients with a hip fracture differs from the total group on several expected differences, such as healthcare utilization that seems to be more specific for a hip fracture patient and the higher costs that accompany them.. However, even though the two subsamples might not be one-on-one comparable, the issue raised for the total group of chronically ill older adult patients still applies to the group admitted due to a hip fracture, where still a proportion of patients does not receive aftercare in the form of GR, CN, ST or LTC, although to a lesser extent.

### Strengths and limitations

Because we used data from one of the largest Insurance companies in the Netherlands with a 30% market share, we were able to analyze a large and representative group of the older adults in the Netherlands. Also, the fact that the company not only collected claims data on a DRG level but on healthcare activity level means that we can actually analyze the chain of healthcare in much greater detail. We were unable to include claims made under the social support act. This is healthcare reimbursed by municipalities which focuses on providing assistance in daily living (for instance grocery or meal services or cleaning support) in order to let the older adult live independently for as long as possible.

In 2015, an average of 22% of all persons aged 75 and over resided in a long-term care facility which is similar to but slightly higher than the 18% of the older adults we identified residing in nursing homes. Our inclusion criterion ‘using medications from at least two different pharmacy-based cost groups’ most likely lead to an under-sampling of older adults residing in long-term care facilities. This is because in those facilities, the medication costs are directly claimed with the facility and not with the insurance company. Also, by using pharmacy-based cost groups, not all drugs administered in the inpatient setting could be used to identify morbidities of the FE. These two reasons lead to a lower morbidity rate for cancer and dementia. This does not mean that a large part of our subjects does not suffer from these diseases; it means that we were unable to identify them.

## Conclusions

The organization of post-discharge healthcare for chronically ill older adult patients may not be organized sufficiently to guarantee appropriate care to restore functional activity. Although receiving aftercare is not a clear predictor of readmissions in our study, the results do seem to indicate that older adults receiving community nursing in the first 90 days less often die compared to chronically ill older adult patients with other types of aftercare or no aftercare. Therefore, an area for improvement could be the deployment of a home nurse after hospital admission for these patients in order to bridge the transfer from hospital to the primary care setting. More research is however necessary to examine predictors of readmissions and mortality in both older adult patients discharged from hospital.

## Data Availability

The data that support the findings of this study are available from insurance company Zilveren Kruis Achmea, but restrictions apply to the availability of these data, which were used under license for the current study, and so are not publicly available. Data are however available from the authors upon reasonable request and with permission of Zilveren Kruis Achmea.

## Abbreviations

CN: Community nursing
ER: Emergency room
GP: General practitioner
GR: Geriatric rehabilitation
HCI: Healthcare intensity
ICU: Intensive care unit
LRT: Lower respiratory tract
LTC: Long-term care
PAOD: Peripheral arterial occlusive disease
SCP: Social and Cultural Planning Office
SES: Socioeconomic status
ST: Short stay
